# Regulation of TGFβ/SMAD signaling by long non-coding RNAs in different cancers: Dark Knight in the Castle of molecular oncology

**DOI:** 10.1016/j.ncrna.2020.12.003

**Published:** 2021-01-07

**Authors:** Aima Adylova, Auyezova Ardak Mukhanbetzhanovna, Rukset Attar, Ishmuratova Margarita Yulaevna, Ammad Ahmad Farooqi

**Affiliations:** a Biomedical Engineering & Molecular Medicine PhD candidate, Guangdong Key Laboratory for Genome Stability & Disease Prevention and Carson International Cancer Center, Shenzhen University School of Medicine, Shenzhen, Guangdong, 518060, China; b Department of Healthcare Management, KSPH Kazakhstan Medical University, Almaty, Kazakhstan; c Department of Obstetrics and Gynecology, Yeditepe University, Turkey; d E. A. Buketov Karaganda University, Kazakhstan; e Institute of Biomedical and Genetic Engineering (IBGE), Islamabad, Pakistan

**Keywords:** Cancer, Oncology, Carcinogenesis, Signaling, Non-coding RNAs

## Abstract

One of the complex themes in recent years has been the multi-layered regulation of TGFβ signaling in cancer cells. TGFβ/SMAD signaling pathway is a highly complicated web of proteins which work spatio-temporally to regulate multiple steps of carcinogenesis. TGFβ/SMAD has been shown to dualistically regulate cancer progression. Therefore, TGFβ/SMAD signaling behaves as a “double-edged sword” in molecular oncology. Accordingly, regulation of TGFβ/SMAD is multi-layered because of oncogenic and tumor suppressor long non-coding RNAs (LncRNAs). In this review, we have summarized most recent breakthroughs in our understanding related to regulation of TGFβ/SMAD signaling by lncRNAs. We have comprehensively analyzed how different lncRNAs positively and negatively regulate TGFβ/SMAD signaling in different cancers. We have gathered missing pieces of an incomplete jig-saw puzzle of lncRNA-interactome ranging from “sponge effects” of lncRNAs to mechanistic modulation of TGFβ/SMAD signaling by lncRNAs.

## 1 Introduction

Transforming growth factor-β (TGFβ) signaling is mediated by TGFβ ligands which transduce the signals to the nucleus through the action of SMADs [1]. Studies have shown that different TGFβ members act through specified SMADs. Numerous members of the TGFβ family initialize signaling by formation of a membrane receptor complex known as heterotetramerization. In these complexes, type II receptor subunits cause phosphorylation and activation of type I receptor subunits for the transmission of the signals by phosphorylated-SMAD proteins. Normally, in the basal state, SMAD proteins remained in the cytoplasm but after phosphorylation, SMADs moved to the nucleus and controlled the transcription.

First, we will provide an overview of the molecular players that are versatile regulators of the core pathway. Previous studies had shown that type II receptor phosphorylated type I receptor at various serine and threonine residues in the juxtamembrane domain. Importantly, this domain is also rich in glycine and serine. Phosphorylation of the glycine and serine residues enabled the attachment of the R-SMADs (receptor-regulated SMADs) [2,3]. SMAD1, SMAD2, SMAD3, SMAD5 and SMAD8 are R-SMADs. Type I receptor phosphorylated R-SMADs on two serine residues. Phosphorylation of R-SMADs allowed them to form complexes with the common mediator SMAD4. Accordingly, SMAD complexes moved into the nucleus to transcriptionally regulate target gene networks [4,5]. TGFβ/SMAD signaling is tightly controlled by negative regulators. Negative feedbacks in the TGFβ superfamily signaling cascades are modulated by activation of the I-SMADs (inhibitory SMADs). Different I-SMADs (SMAD6 and SMAD7) have been characterized. SMAD7 interacted constitutively with the ubiquitin ligases. SMURF1 and SMURF2 are HECT-domain E3 ubiquitin ligases which ubiquitinate and degrade SMADs and TGF receptors [[6], [7], [8], [9]].

Genomes are extensively transcribed and give rise to thousands of lncRNAs (long non-coding RNAs), which are defined as RNAs longer than 200 nucleotides that are not translated into functionally active proteins. Excitingly, this broader definition encompassed a large and highly heterogeneous collection of transcripts that differed in their genomic origin and biogenesis [[10], [11], [12], [13]].

Based on the scintillating insights gained from decades of research, it is becoming gradually more understandable that TGFβ/SMAD pathway is contextually regulated by non-coding RNAs in different cancers. We have partitioned this review into different sections to exclusively focus on the interplay between SMADs and lncRNAs.

## 2 Regulation of SMAD2/3 by LncRNAs

It has been shown that co-culture of mesenchymal stem cells-conditioned medium and gastric cancer cells induced chemoresistance and stemness in gastric cancer cells [14]. Importantly, levels of p-TGFβRI, p-TGFβRII, p-SMAD2 and p-SMAD3 were found to be enhanced when gastric cancer cells were co-cultured with MSCs. These findings suggested that TGFβ1 secreted by MSCs contributed to drug-resistance through binding to TGFβ receptors and activation of SMAD2 and SMAD3. TGFβ1 induced upregulation of MACC1-AS1. Furthermore, TGFβ1 inhibitors and TGFβRI inhibitors abrogated MACC1-AS1 expression in gastric cancer cells treated with conditioned medium of MSCs. CPT1 (carnitine palmitoyltransferase 1) played critical role in chemoresistance. MACC1-AS1 directly interacted with miR-145-5p. Intriguingly, binding sites between CPT1 and miR-145-5p were not identified. However, still, miR-145-5p was noted to inhibit the expression of CPT1. When MACC1-AS1 and miR-145-5p were co-transfected in gastric cancer cells, miR-145-5p caused partial reversal of MACC1-AS1-mediated increase in the expression of CPT1, which indicated that these molecules are connected through yet unidentified regulatory networks [14].

MIR100HG is a lncRNA and a tricistronic host gene of miR-100, miR-125b and let-7a [15]. MIR100HG was significantly upregulated by TGFβ. TGFβ activated SMAD2/3, which thus regulated the transcription of MIR100HG and subsequently increased miR-125b and miR-100 levels, thus promoting EMT and tumorigenesis in PDAC cells. Paradoxically, let-7 family members have the ability to inhibit tumorigenesis in PDAC. Therefore, astonishingly, TGFβ induced upregulation of miR-100 and miR-125b but simultaneously repressed let-7a. LIN28B upregulation has been reported to reduce the levels of let-7 and increase let-7 targets during the progression of PDAC. let-7a levels were noted to be increased in LIN28B-silenced PANC-1 cells. S2-007 cells with impaired miR-125b activity had noticeably reduced metastatic spread and colonization to the liver in tumor bearing mice [15].

miR-665 acted as a tumor suppressor miRNA and directly targeted TGFBR1 and TGFBR2 [16]. Linc00462 effectively blocked miR-665-mediated targeting of TGFBR1 and TGFBR2. Overexpression of linc00462 significantly enhanced the levels of p-SMAD2 and p-SMAD3. However, overexpression of miR-665 significantly reduced the levels of p-SMAD2 and p-SMAD3. Mice injected with linc00462 expressing PANC-1 cells metastasized more efficiently in tumor bearing mice [16].

**Fig. 1:**
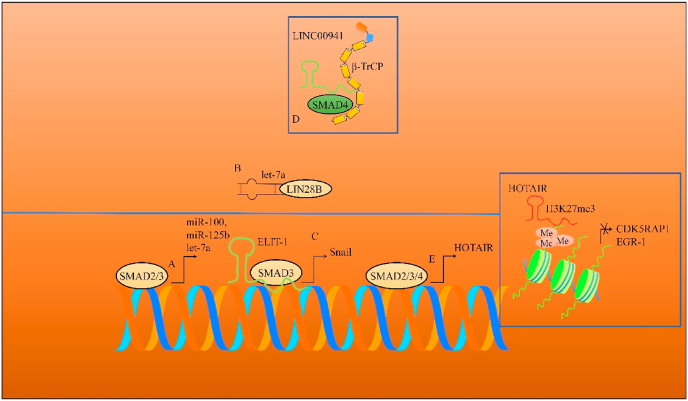
(A–B) MIR100HG is a lncRNA and a tricistronic host gene of miR-100, miR-125b and let-7a. SMAD2/3 transcriptionally upregulated miR-100 and miR-125b. let-7a is a tumor suppressor but LIN28B interfered with the processing of let-7a. (C) ELIT-1 worked synchronously with SMAD3 and upregulated Snail. (D) LINC00941 protected SMAD4 from degradation by β-TRCP. (E) SMAD2/3/4 stimulated the expression of HOTAIR. HOTAIR epigenetically inactivated CDK5RAP1 and EGR-1.

EMT-associated lncRNA induced by TGFβ1 (ELIT-1) physically interacted with SMAD3 and potentiated SMAD3-mediated transcriptional upregulation of Snail (shown in Fig. 1) [17]. Collectively, these findings supported the notion that ELIT-1 worked synchronously with SMAD3 and stimulated the expression of target genes for epithelial-mesenchymal transition.

Cancer associated fibroblasts (CAFs) have a crucial role in the tumor microenvironment. TGFβ1 derived from CAFs caused EMT in breast cancer cells. TGFβ1-mediated activation of SMAD2/3/4 induced transcriptional upregulation of HOTAIR in breast cancer cells [18]. HOTAIR promoted H3K27-mediated trimethylation of the CDK5RAP1 and EGR-1 promoters, thereby causing the activation of CDK5 [18]. Overall, these findings highlighted that CAFs secreted TGFβ1 which triggered HOTAIR upregulation in breast cancer cells. HOTAIR and EZH2 induced H3K27-mediated trimethylation of the EGR-1 and CDK5RAP1promoters (shown in Fig. 1).

## 3 MIR22HG

**Fig. 2:**
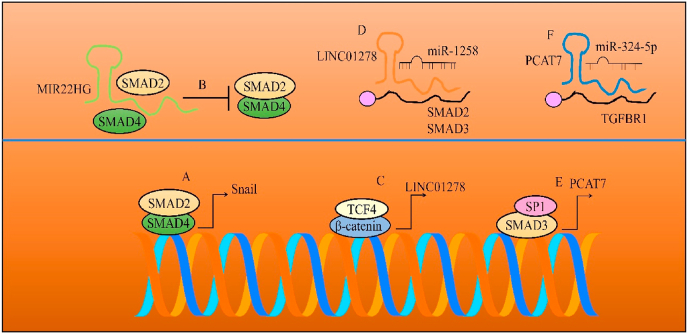
(A–B) SMAD2 and SMAD4 induced the expression of Snail. However, MIR22HG interfered with the heterodimerization of SMAD2 and SMAD4. (C–D) TCF4/LEF1 binding site was identified in promoter region of LINC01278. β-catenin and TCF4 transcriptionally upregulated LINC01278. LINC01278 interfered with miR-1258-mediated targeting of SMAD2 and SMAD3. (E–F) SP1 and SMAD3 stimulated the expression of PCST7. PCAT7 promoted TGF/TGFR signaling via potentiating the expression of TGFBR1.

MIR22HG exerted its tumor suppressive effects through competitively binding to SMAD2 and blocking the association between SMAD4 and SMAD2 in colorectal cancer cells (shown in Fig. 2) [19]. SMAD2/SMAD4 transcriptionally stimulated the expression of Snail. SNAIL is a repressor protein that promoted EMT. Tumor xenografts derived from MIR22HG-overexpressing cells were found to be markedly. Furthermore, number of metastatic nodules were significantly reduced in mice inoculated with MIR22HG-overexpressing cancer cells. Additionally, metastatic foci were sparse and small in tumor bearing mice [19].

## 4 LINC01278

TCF4/LEF1 binding site was identified in promoter region of LINC01278 [20]. Binding of TCF4 to LINC01278 promoter was noted to be increased upon overexpression of β-catenin. Additionally, β-catenin overexpression caused a significant increase in LINC01278 expression in HCC cells. LINC01278 promoted TGF/SMAD signaling by interfering with miR-1258-mediated targeting of SMAD2 and SMAD3 (shown in Fig. 2). Lung metastasis was reduced in mice xenografted with LINC01278-silenced SMMC-7721 [20].

## 5 PCAT7

PCAT7 interfered with miR-324-5p-mediated targeting of TGFBR1 [21]. Activation of TGFβ/SMAD signaling promoted cancer. Chromatin immunoprecipitation assays provided evidence of the enrichment of SP1 and SMAD3 on the promoter of PCAT7 (shown in Fig. 2). TGFβ‐induced SMAD3/SP1 complex not only increased the DNA‐binding affinity of SP1 but also promoted the binding of SP1 to the promoter region of PCAT7. Bone metastasizing capacity of PCAT7-overexpressing prostate cancer cells was found to be notably enhanced in tumor bearing mice. PCAT7 overexpression accelerated the onset of bone metastasis in tumor bearing mice [21].

## 6 CASC9

CASC9 was found to be frequently overexpressed in colorectal cancers [22]. CPSF3 is a CASC9-interacting protein. CPSF3 knockdown significantly reduced proliferation ability of HCT-116 and SW620 cells. Decay of TGFβ2 mRNA was rapid in CPSF3-silenced or CASC9-depleted cancer cells. TGFβ2 and p-SMAD3 levels were reduced in CASC9-knockdown cells, whereas their levels were increased in CASC9 overexpressing colorectal cancer cells [22].

## 7 lnc-TSI

lnc-TSI reduced invasive and metastasizing potential of ccRCC cells [23]. Mechanistically, it was shown that lnc-TSI interacted with the MH2 domain of SMAD3 and blocked the association between TGFβ receptor I and SMAD3 in ccRCC cells. Metastatic lesions were considerably enhanced in mice inoculated with lnc-TSI-silenced Caki-1 cells, whereas lnc-TSI overexpression markedly reduced the metastatic lesions in tumor bearing mice [23].

## 8 PVT1

PVT1 effectively blocked miR-140-5p-mediated targeting of SMAD3 in cervical cancer cells. PVT1 promoted the proliferation and metastasis by potentiating the expression of SMAD3 [24].

Moreover, PVT1 knockdown reduced the levels of mesenchymal markers including N-cadherin, Snail and Slug, while it increased epithelial marker expression of E-cadherin. PVT1 knockdown also efficiently inhibited TGFβ/SMAD signaling [25].

## 9 ANRIL

There was a marked reduction in the levels of TGFβ1 and p-SMAD2 in ANRIL knockdown prostate cancer cells [26]. ANRIL knockdown significantly enhanced the levels of let-7a and p-SMAD7. Collectively, these findings suggested that ANRIL inhibition simultaneously potentiated the expression of let-7a and caused inactivation of TGFβ/SMAD signaling. It was interesting to note that inhibition of let-7a potently enhanced the invasive potential of ANRIL-silenced prostate cancer cells [26].

## 10 SPRY4-IT

SPRY4-IT played contributory role in the progression of thyroid cancer. TGFβ1 and p-SMAD2/p-SMAD3 levels were noted to be enhanced in SPRY4-IT-overexpressing thyroid cancer cells [27].

## 11 SMAD2-mediated upregulation of oncogenic LncRNAs

SMAD2 has been shown to trigger the upregulation of various lncRNAs to promote cancer. In this section, we will exclusively focus on the different lncRNAs reportedly stimulated by SMAD2.

SMAD2 transcriptionally upregulated LINC00266-1 in osteosarcoma cells [28]. Invasive potential of LINC00266-1-silenced osteosarcoma cells was found to be notably reduced. LINC00266-1 knockdown resulted in a notable reduction in the levels of SMAD2. miR-548c-3p negatively regulated SMAD2 in osteosarcoma cells. However, LINC00266-1 blocked miR-548c-3p-mediated targeting of SMAD2 [28].

SMAD2 directly bound to the promoter of MACC1-AS1 and stimulated its expression [29]. Consequently, MACC1-AS1 interfered with miR-145-mediated targeting of SMAD2 in nasopharyngeal carcinoma cells. miR-145 effectively inhibited cancer progression but MACC1-AS1 antagonized its cancer suppressive effects [29].

## 12 LncRNA mediated upregulation of SMAD2 to promote carcinogenesis

There are direct pieces of evidence related to positive regulation of SMAD2 by lncRNAs to promote cancer. Seemingly, SMAD2 and lncRNAs promote each other to enhance proliferation and metastasizing ability of cancer cells. In this section we will summarize how different lncRNAs potentiated SMAD2 expression.

NEAT1 interfered with miR-200b-3p-mediated targeting of SMAD2. NEAT1 and SMAD2 promoted the proliferation and invasion of melanoma cells. However, these effects were reversed by upregulation of miR-200b-3p [30].

There was a noteworthy decrease in the migration, invasion and stemness of HOXA-AS2-silenced bladder cancer cells [31]. Overexpression of miR-125b and HOXA-AS2 knockdown significantly lowered the levels of SMAD2 in T24 cells. HOXA-AS2 stimulated the expression of SMAD2 by blockade of miR-125b-mediated targeting of SMAD2 [31].

miR-132-3p directly targeted SMAD2 and suppressed cancer progression [32]. However, ILF3-AS1 antagonized miR-132-3p-mediated cancer suppressive effects. ILF3-AS1 inhibition caused marked reduction in SMAD2 levels in tumor tissues, whereas miR-132-3p expression was upregulated in xenografted mice [32].

## 13 Positive role of SMAD2/3 in cancer suppression

Apart from tumor promoting role of SMAD2 and SMAD3, emerging evidence revealed that TGFβ/SMAD signaling played central role in the induction of apoptosis. ANRIL silencing induced activation of SMAD-driven signaling [33]. Phosphorylated levels of SMAD1, SMAD2 and SMAD3 were noted to be enhanced in ANRIL-silenced Burkitt lymphoma cells. Use of LY2109761 (TGFβ receptor inhibitor) abrogated apoptotic cell death in ANRIL-silenced Burkitt lymphoma cells [33].

Overexpression of TFAP2A-AS1 caused apoptosis in MCF-7 and MDA-MB-231 cells [34]. TFAP2A-AS1-interfered with miR-933-mediated targeting of SMAD2 in breast cancer cells. Tumor volumes and weights were significantly smaller in mice injected with TFAP2A-AS1-overexpressing MCF-7 cells [34].

## 14 SMAD4

***Oncogenic role of SMAD4:*** SMAD4 has been shown to play oncogenic role in different cancers. In this section, we will discuss how SMAD4 strategically worked with oncogenic LncRNAs to promote cancer. However, SMAD4 also inhibited tumor suppressor lncRNAs.

LINC00941 is a binding partner of SMAD4. LINC00941 inhibition lowered the levels of SMAD4, whereas overexpression of LINC00941 efficiently enhanced SMAD4 levels [35]. SMAD4 is polyubiquitinated and degraded by β-TrCP. Binding of β-TrCP and SMAD4 was noted to be enhanced in LINC00941-silenced cancer cells but conversely, LINC00941 overexpression significantly inhibited the binding of β-TrCP and SMAD4. LINC00941 interacted with MH2 domain of SMAD4 and blocked the binding of β-TrCP to the SMAD4. It was shown that mice inoculated with LINC00941-silenced LoVo cells developed fewer lung metastatic nodules. Whereas, mice injected with LINC00941-overexpressing HCT116 cells generated notably higher number of lung metastatic nodules [35].

LncRNA in nonhomologous end joining (NHEJ) pathway 1 (LINP1) is a tumor suppressor lncRNA [36]. However, TGF transcriptionally downregulated LINP1 to promote metastasis. SMAD4 transcriptionally silenced LINP1. LINP1 knockdown significantly downregulated E-cadherin and simultaneously upregulated expression of SNAIL and N-cadherin in A549 cells. However, LINP1 overexpression led to an increase in E-cadherin and marked decline in the levels of SNAIL and N-cadherin [36].

miR-34a is a tumor suppressor and negatively regulates SMAD4 [37]. However, Lnc34a worked synchronously with DNMT3a, HDAC1 and PHB2 for the epigenetic inactivation of miR-34a. miR-34a overexpression significantly inhibited bone metastasis, whereas overexpression of SMAD4 not only increased the invasion of cancer cells to the bones and but also accelerated bone damage [37].

SMAD4 played critical role in the proliferation and metastasis of osteosarcoma cells. Different oncogenic lncRNAs promoted the expression of SMAD4 to enhance proliferation capacity of osteosarcoma cells. MALAT1 interfered with miR-205-mediated targeting of SMAD4 and promoted the proliferation of MG-63 and SAOS-2 cells [38].

SNHG7 also effectively blocked miR-34a-mediated targeting of SMAD4 to enhance metastasizing potential of osteosarcoma cells [39]. There was a marked reduction in the growth of tumors in mice xenografted with SNHG7-silenced MG63 cells [39].

***Tumor Suppressor Role of SMAD4:*** AWPPH worked synchronously with EZH2 and epigenetically inactivated SMAD4 to promote proliferation and migration of bladder cancer cells [40].

## 15 SMAD5

SMAD5-AS1 is an oncogenic lncRNA having central role in carcinogenesis. SMAD5-AS1 is noted to potentiate the expression of SMAD5 by blocking miRNA-mediated targeting of SMAD5 in nasopharyngeal carcinoma cells. SMAD5-AS1 promoted EMT by interfering with miR-106a-5p and miR-195-mediated targeting of SMAD5 [41,42]. Tumorigenesis was inhibited either through knockdown of SMAD5‐AS1 or miR‐106a‐5p upregulation in tumor bearing mice [41]. LINC01410 effectively inhibited miR-124-3p-mediated targeting of SMAD5 in HuCCT1 and RBE cells [43].

MALAT1 blocked miR-142-3p-mediated inhibition of SMAD5 [44]. MALAT1 depletion caused considerable reduction of tumor growth in tumor bearing mice, while the ectopic expression of MALAT1 significantly promoted tumor growth. Additionally, expression levels of E-cadherin and miR-142-3p were noted to be significantly upregulated in the MALAT1 knockdown groups [44].

## 16 SMAD7

***SMAD7 as a Tumor Suppressor: Positive regulation of SMAD7 by LncRNAs:*** NFκB1 transcriptionally downregulated DGCR5 in glioma cells [45]. NFκB1 overexpression caused significant downregulation of DGCR5, whereas NFκB1 silencing promoted the expression of DGCR5. DGCR5 overexpression remarkably increased SMAD7 levels in U251-MG and SHG44 cells. DGCR5 effectively sequestered miR-21 and miR-23a to exert cancer suppressive effects. SMAD7 was noted to be directly targeted by miR-21. Likewise, PTEN is a tumor suppressor phosphatase notably involved in the inactivation of PI3K/AKT-driven signaling. miR-23a negatively regulated PTEN and enhanced tumor progression but DGCR5 interfered with miR-23a-mediated inhibition of PTEN. DGCR5 overexpression considerably reduced the tumor volumes and weights of SHG44-derived and U251-MG-derived tumors. Furthermore, overexpression of DGCR5 significantly increased the level of the epithelial marker E-cadherin in tumor tissues [45].

A1BG-AS1 also acted as a tumor suppressor lncRNA and potentiated the expression of PTEN and SMAD7 in HepG2 cells [46]. miR-216a-5p has been shown to target SMAD7 and PTEN and enhance tumor progression. A1BG-AS1 blocked miR-216a-5p-mediated inhibition of PTEN and SMAD7 [46].

X-inactive specific transcript (XIST) acted as a sponge and protected SMAD7 from targeting by miR-92b [47]. Metastasis rate was significantly higher in mice inoculated with miR-92b-overexpressing SMMC-7721 cells [47].

LINC00968 enhanced SMAD7 expression by sequestering the miR-21-5p away [48]. There was a marked reduction in the metastatic foci on the lung surface of mice injected with LINC00968-overexpressing A549 cells [48].

***Negative regulation of SMAD7:*** SNAI3‐AS1 enhanced HCC tumorigenesis by interacting with UPF1 [49]. UPF1 is involved in nonsense‐mediated mRNA degradation of different proteins. It has previously been revealed that UPF1 triggered the degradation of SMAD7 [50]. SNAI3‐AS1 and UPF1 worked synergistically and promoted TGF/SMAD signaling. SNAI3‐AS1 knockdown significantly decreased the levels of p-SMAD2 and p-SMAD3 [49].

## 17 SMURF1 and SMURF2

***Regulation of SMURFs by LncRNAs:*** Anaplastic lymphoma kinase (ALK) is a receptor tyrosine kinase and it has been shown that translocations or deregulated expression of ALK contribute to carcinogenesis. ALK^negative^ ALCL (anaplastic large cell lymphoma) is a malignant form of non-Hodgkin lymphoma. MIR503HG, an lncRNA has been found to be highly expressed in ALK-negative ALCL [51]. MIR503HG depletion effectively suppressed proliferation capacity of ALK-negative ALCL cells. There was a marked reduction in tumor formation in the mice inoculated with MIR503HG-depleted MAC-1 cells. Inactivation of MIR503HG simultaneously repressed the expression of miR-503. SMURF2 is directly targeted by miR-503. SMURF2 induced ubiquitination-dependent and proteasome-mediated degradation of TGFBR [51]. MIR503HG promoted the proliferation of ALK^negative^ALCL cells through the miR-503/SMURF2/TGFBR signaling axis.

SNHG3 blocked miR‐577-mediated targeting of SMURF1 [52]. SMURF1 gain of function enhanced proliferation capacity of SNHG3-silenced PC3 cells. Tumor weights and volumes were noted to be drastically reduced in mice xenografted with SNHG3-silenced prostate cancer cells [52].

## 18 Concluding remarks

In this review we have attempted to provide a summary of the latest advancements in our understanding related to regulation of TGFβ/SMAD signaling by lncRNAs in different cancers. Nonetheless, this knowledge still represents only a small fraction of the landscape of their gene regulatory potential. Keeping in view their hallmark features and pleiotropic roles, disease-related lncRNAs will expectedly gain greater relevance and significance in the context of individualized medicine. Advancements in this dimension will go hand in hand with better understanding and comprehensive knowledge of the gene regulation modalities of lncRNAs.

## Declaration of competing interest

I declare on the behalf of all authors that none of the authors have any conflict of interest.
